# Superior monocular visual function but compromised binocular balance in precision shooters compared to age and refraction matched controls

**DOI:** 10.1038/s41598-025-14497-9

**Published:** 2025-08-06

**Authors:** Izabela K. Garaszczuk, Wiktoria Jenczewska, Magdalena Asejczyk

**Affiliations:** Visual Optics Group, Department of Optics and Photonics, Wroclaw University of Science and Technology (Politechnika Wrocławska), wyb. Wyspianskiego 27, 50-370 Wrocław, Poland

**Keywords:** Medical research, Outcomes research

## Abstract

Shooting sports demand exceptional visual performance, yet detailed assessments of visual function in precision shooters remain limited. This cross-sectional study evaluated 28 pistol and rifle shooters and 20 age- and refractive-error-matched non-athletic controls. Participants underwent comprehensive visual assessments, including tests of visual acuity (VA), Vernier acuity, contrast sensitivity, binocular vision, accommodation, ocular biometry, perimetry, and eye movement tracking. A subgroup of national-level athletes was also analyzed. Compared to controls, shooters demonstrated superior near VA (−0.08 ± 0.06 vs. 0.03 ± 0.07 logMAR; *p* = 0.003), binocular Vernier acuity (5.4 ± 3.2 vs. 8.7 ± 5.1 arcsec; *p* = 0.032), and dominant eye contrast sensitivity (*p* = 0.005). National-level shooters showed fewer gaze shifts (*p* = 0.044), more stable fixation, and better stereoacuity (25 vs. 35 arcsec; *p* = 0.005). Modality-specific differences were observed: pistol shooters exhibited better distance acuity and central field sensitivity, while rifle shooters—despite being older—performed better in near VA. However, covering one eye to avoid diplopia, which is inherent in precision shooting, may cause suppression of the covered eye when performed frequently and for prolonged periods. This ultimately may explain why shooting experience correlates with reduced binocular balance and a worse near point of convergence (*r* = 0.335, *p* = 0.020). These findings suggest that visual expertise in precision shooting is linked to task-specific visual adaptations. Tailored visual training programs may enhance performance and mitigate training-induced imbalances.

## Introduction

Visual performance can be characterized by different parameters, such as visual acuity, accommodation amplitude, binocular vision parameters or visual reaction time. Research indicates that athletes have higher visual skills than their non-athletic peers and these skills may be determined by the type of physical activity they perform^1,2^. In this context, some have suggested the importance of monitoring visual skills during training periods to increase performance^3,4^. In targeted sports such as golf, archery and precision shooting, visual information and subsequent processing seem to be essential^5^. These sports are self-paced and generally require a great deal of accuracy and ability to maintain the *quiet eye* state^5–7^. Specifically, in Olympic shooting, the movement precision and visual processing must be at a maximum level^8^. The target is located 10 m away and the diameter of the 10 points area is 11.5 ± 0.1 mm in pistol and 0.5 ± 0.1 mm in rifle shooting^9^ so that minimal angular errors (0.016° for rifle^10^ or 0.066° for pistol shooting^11^) can prevent a shooter from achieving the maximum score^12^. Practitioners emphasize the importance of visual acuity during aiming^13,14^. Additionally, the stability of the arm and weapon set, hand-eye coordination,^15^ as well as the ability to clearly perceive the sights of the gun, are important in sports of such extreme precision. These parameters depend on adequate levels of accommodation to be maintained for longer periods of time^16^. Precision shooters not only have to be able focus their gaze on the front sight^17^ but also to maintain this focus for longer periods of time and also be able to alternate their gaze between different distances before the shot, which requires good accommodation facility^18^. The study of Carkeet et al. emphasized the importance of visual acuity at near, and accommodation relaxation in pistol shooting^19^. Accommodation is of such importance in aiming action that some practitioners recommend prescribing small near vision correction even for non-presbyopic shooters to aid their accommodation.

Understanding athletes’ unique visual demands can guide targeted interventions to enhance performance,^17,20^ reduce fatigue, and preserve long-term visual health. Despite all these visual skills that may influence marksmanship, the literature on precision shooters’ visual skills is lacking. This could mean that athletes do not optimize their potential. Only a few studies show that shooting training affects basic visual skills (accommodation and visual acuity) depending on the level of experience; however, these effects were observed only directly after the competition^21^. Other studies report higher visual acuity in archers^22^. Beside that, most sport vision studies focus on interceptive sports, such as football or hockey, and expert superiority in shooting was studied only in clay target shooters in the simple reaction time. Clay skeet shooters, however, perform binocularly, while in static modalities blinders are used to avoid diplopia. Furthermore, there exists a risk of suppression of the occluded eye during prolonged and frequent training periods, which can have detrimental effect on binocular vision. Correcting for intermittent central suppression has been shown to improve marksmanship^23^. Further research on shooting physiology should be conducted and contain more complex analyses to investigate the multifaceted processes associated with different performance determinants in Olympic shooting^8^. This study aims to fill this gap in knowledge by comparing a broad range of visual parameters in precision rifle and pistol shooters with controls.

## Methods

The study methodology was approved by the Research Ethics of Wroclaw University of Science and Technology (approval number: O-22-42) adheres to the tenets of the Declaration of Helsinki. Informed consent was obtained from each participant (or their legal guardians) after explaining the nature of the study. The study included forty-eight participants − 28 shooters (16 pistol shooters and 12 rifle shooters) of varying experience and 20 age-matched controls. The control group consisted of healthy, nonathletic volunteers with no previous shooting experience. Data collection was conducted from November 2022 to November 2024. Shooters specialized in precision air rifle or pistol shooting were recruited from members of the shooting section of various Sports Clubs in Poland, including *Silesian Military Sports Club* based in Wrocław (Poland) and from the *Polish National Shooting Team* (15 participants). All shooters in the group were actively competing in shooting sports at a minimum of the national level. Participants were recruited from multiple clubs and teams, ensuring diversity and minimizing selection bias until the calculated sample size was reached. Sample size was estimated using the assumptions for standard normal distribution with a 5% level of significance, power of 90%, and standard deviation of Vernier acuity of 6.3‘’. A sample size (n) for which a 50% decrease/increase in Vernier acuity could be discriminated at 5% level of significance with 90% power was estimated at *n* ≥ 41. Exclusion criteria included high refractive error (> 6 diopters), visual acuity of less than 1.0 logMAR and substantial changes in the visual field of less than 20 degrees radius. The latter two parameters are arbitrarily used as an eligibility criterion for Paralympic shooting^24^. Participants wore their visual corrections for testing and, if necessary, a new refractive error correction was prescribed. All participants were tested under the same environmental conditions. Assessors were not blinded to participants’ shooting experience, however automated measurement tools were used wherever applicable to reduce observer bias. Refractive error was measured objectively using the WAM 5500 open-field autorefractor system subjective refinement by an optometrist was performed. Cycloplegia was not used due to the adult, competitive athlete sample. Habitual correction with glasses, refined by an optometrist, was required for both distant and near tasks, described further. Subjective refraction was performed to ensure that the subjects met the inclusion criteria and had no strabismus or binocular vision problems.

### Eye dominance and hand dominance

The participant’s sighting eye dominance was determined using the ‘hole in hand’ variation of the Miles test.

The convergence near-point test was used to establish eye dominance for near viewing. To assess handedness, the Edinburgh Handedness Inventory Short Form was used^25^. Athletes were additionally asked which hand they use to pull the trigger. Additionally, a fogging technique with a + 2.00 D lens was used to check for eye dominance during subjective refraction procedures. This latter test reflected the results of the Miles test.

### Monocular and binocular visual acuity at distance and near

The Freiburg Visual Acuity test (FrACT)^26^ was used to assess best corrected visual acuity (BCVA) for distant vision with the *Acuity Assessment Sloan Letters* option. The monocular visual acuity (BCVA OD and BCVA OS, for right and left eye, respectively) and best corrected binocular visual acuity (BCVA OU) expressed in logMAR scale were evaluated. The calibration and luminance of the room was ensured according to the checklist provided by the software developer. Optotypes were displayed at 5 m on a 15.6 in. laptop screen (Lenovo Ideapad L340) with a resolution of 1920 × 1080 pixels. The screen brightness was set to its maximum level, with the battery-saving mode turned off. The screen was turned on at the beginning of the session to reach its maximum luminance before commencing measurements. Participants were required to type the letters they see on the screen with the remote QWERTY keyboard. Measurements terminate after a fixed number of trials (*n* = 30). A logarithmic chart was used to assess near visual acuity monocularly (NVA OD and NVA OS for the right and left eye, respectively) and binocularly (NVA OU) at 40 cm. Habitual correction was required for both distant and near tasks.

### Vernier visual acuity

Vernier acuity measures the ability to detect a misalignment or positional offset between visual stimuli, for example, between two vertical lines when reading a Vernier scale. When aligning front sight and rear sight with the target, athletes perform the Vernier alignment tasks. Vernier acuity is heavily reliant on cortical processing, thus making it a useful indicator of cortical visual function^27^. The *Hyperacuity Vernier Assessment* option of FrACT was used to measure Vernier acuity^26^ monocularly and binocularly at 5 m and results were displayed in seconds of arc (arcsec). Participants performed a two-alternative forced-choice Vernier acuity task, in which they judged whether the upper line was shifted to the left or right relative to the lower line and indicated their response by pressing the corresponding cursor key. Because the task has only two response options, a larger number of trials (*n* = 50) was required to ensure a reliable estimate of the Vernier threshold and to minimize the influence of guessing.

### Horizontal heterophorias

Dissociated heterophoria is the misalignment of the eyes relative to a target in the absence of fusional vergence. An exophoria is a divergent misalignment, whereas an esophoria is a misalignment in the convergent direction. Minimal to no misalignment (< 2 prism diopters) is defined as orthophoria. Occluding one eye (as in shooting training) removes the disparity cue used by fusional vergence to correct this misalignment and results in a drift to the heterophoria position for the relevant viewing distance.^28^.

Penlight was used as a fixation target. To remove the disparity cue, the red Maddox rod was slotted over the right eye of each participant for testing, so the right eye was perceiving the penlight as a red streak. Participants viewed the penlight from 40 cm to 500 cm away to measure their near and distance horizontal heterophoria, respectively. A cross with a scale marking the deviations in prism diopters was used for distance measurements, and for near measurements, a modified Thorington chart was viewed. Participants were instructed to look at the penlight and tell the practitioner the location of the red streak relative to the penlight. To determine the level of phoria in prism diopters [Δ], participants were asked to report the number on the scale closest to the read streak. If the vertical streak passed through a number on the right or on the left side of the penlight, esophoria or exophoria was recorded, respectively. Orthophoria was recorded when the red streak passed directly through the penlight. Esophoria was recorded as a positive value and exophoria - as negative.

### Stereopsis

Stereoacuity measures the smallest detectable depth difference that can be observed binocularly. The 1000 S Fly-S Stereoacuity Chart (Vision Assessment Corp, USA) was used. This chart contains ten four-circle stereoscopic images to assess stereoacuity from 400 to 20 arcsec. Participants were required to look at the chart through linearly polarized filters and identify which one of the four circles in each set had a different perceived depth.

### Contrast sensitivity

Contrast sensitivity (CS), characterizes aspects of visual function that are not as well captured by clinical visual acuity measurement^29^. It is especially useful in determining visual performance in people with vision impairment^30^. Precision shooters often use optical filters for increased contrast sensitivity and decreased glare. The *Mars Letter Contrast Sensitivity* Test was used to test for peak visual contrast sensitivity^31^. Three forms of the chart were supplied (for left eye, right eye, and binocular testing) containing letters of the same angular size (2.5° at 40 cm) with decreasing contrast (from 0.04 to 1.92 logCS). Participants were required to read the letters from left to right across each line of the chart. Participants were encouraged to guess and instructed to restrict their responses to a specific set of optotypes. This approach was adopted to uphold the assumption that the probability of a correct guess was 1/10. Testing was terminated only when the participant made two consecutive errors or reached the end of the chart. This method ensured that the score reflected what the participants could see rather than what they believed they could see. The log contrast sensitivity (log CS) score was determined by taking the log contrast sensitivity value at the lowest contrast letter identified just before two consecutive incorrect responses and then subtracting a scoring correction for each negative response. Binocular and monocular logCS were measured.

### Near point of accommodation and near point of convergence

The near point of convergence and accommodation were measured with *push-up method*, by using a custom-designed metal ruler with a chin rest, handles, and a movable clip with a test chart^32^. A target used in competitive rifle shooting was attached to the ruler. Participants placed their chin on the chinrest and fixated on a target, which was pushed towards them at a constant speed of 1 cm/s starting at 50 cm away. The participant’s task was to report when the target appeared blurry or double, identifying the near points of accommodation (NPA) and convergence (NPC). Afterwards, the test was moved further away from the participant to assess the position of the near point of renewal (NPR), at which the target seen clearly again. The procedure was repeated three times to check for fatigue and the average values of NPA, NPC and NPR were calculated in centimeters.

### Accommodation facility

Accommodation facility is a measure of the speed of accommodative response. It was assessed using ± 2.00 D flipper lenses while participants viewed a standard near vision chart with high-contrast letters that could be easily read at approximately 0.8–1.0 decimal acuity. The chart was held at a distance of 40 cm. The participant indicated when the target was in focus by saying “clear” at which time the examiner was switching to the other set of lenses. This task continued for 30 s during which the number of switches was counted. The results are expressed in full cycles per minute. Average participants can perform 12–20 complete cycles per minute depending on the procedure, lens power and chart distance^33^.

### Fields of vision

Field of vision was tested using the automated PTS 2000 perimeter (Optopol Technology, Poland).

The device was employing a threshold strategy for precise data, with the intensity of the stimuli adjusted until visibility thresholds were pinpointed with ± 1dB accuracy. This method accurately identified participants’ sensitivity levels and visual field defects. The *30–2 C* visual field, with a higher density of points in the macular region of the retina and a standard stimulus size (III) were chosen. The test adapted exposure and interval times to participants’ reaction speeds, capped at 0.4 s to maintain concentration. A head tracker ensured correct positioning of the chin and blind spots using the Heijl-Krakau Technique. For statistical comparisons, the sensitivity values across test points were calculated for each participant and a median value was calculated for each subgroup of subjects in each point of the visual field. Comparisons were made using appropriate tests as described in the Statistical Analysis section. The average test duration was approximately 5–7 min per eye, depending on the participant’s response speed.

### Eye movements

The PTS 2000 perimeter (Optopol Technology) uses an integrated monocular gaze-tracking module that monitors the tested eye during perimetry and counts gaze errors and loss of fixation on the central target, illustrated by a *Gaze shift diagram* showing the amplitude of eye movements (expressed in arbitrary units) over time. The non-tested eye was covered, in line with standard automated perimetry protocols. The gaze-tracking module operates at a sampling frequency of approximately 25 Hz, with angular resolution sufficient to detect fixation losses and gaze shifts within ± 1°–2° of deviation from the central fixation target.

**Fig. 1:**
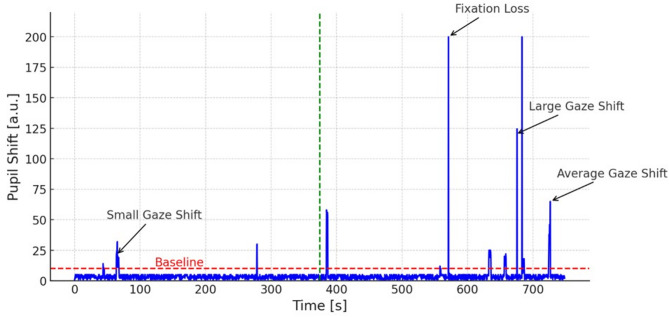
An exemplary gaze shifts graph extracted from the raw perimetric data. The green line divides the data into two halves and the red line denotes the level of small eye movements.

*Gaze Tracker*, set to *Level 2*, records pupil movement and stops or holds the test for eye closures, blinks, or large deviations of eyes from the target, resuming automatically when these situations resolve. It provides the practitioner with a number and amplitude of eye shifts, which are an indication of participants’ fatigue and lowered attention. Eye movements, classified into small movements and large fixation errors, were extracted from raw XML perimetric files. Because the PTS 2000 gaze-tracking module outputs fixation quality in arbitrary units (0–200) without direct angular calibration, gaze shifts were categorized based on manufacturer thresholds: <10 (baseline small fixational movements that occur even when the eye is focused on a static target (baseline) ), 10–50 (small shifts), 50–100 (average shifts), 100–150 (large shifts), and > 150 (fixation losses, which triggers the perimetric device to pause). To aid interpretability, the proportion of total measurement time spent within baseline fixational movement levels versus the time spent above this level, which reflects gaze stability (‘quiet eye’) was calculated. The gaze shifts were divided into small gaze shifts [between 10 and 50], average gaze shifts [50, 100], large gaze shifts [100, 150] and fixation losses [> 150]. An example gaze shift diagram is in (Fig. 1).

The number of movements below 10 (Baseline movements) and above 10 (Gaze shifts) were counted separately. Additionally, small, average, and large gaze shifts and fixation losses were counted for each participant. Since each participant’s measurements time was different, resulting in a different sample size, the number of frames corresponding with each type of eye movement was divided by the total number of frames sampled and expressed as a percentage. Prolonged test time can contribute to participant fatigue and affect gaze stability. To address this, we analyzed gaze shifts in the first and second halves of each test to assess fatigue effects and reported these ratios accordingly. Therefore, the numbers of eye shifts were counted separately for the first and for the second half of perimetric measurements and the ratio of gaze shifts to Baseline movements was counted for each of the halves (Ratio First Half and Ratio Second Half, respectively).

### Ocular biometry

Some parameters assessed in this study highly depend on refractive error, therefore ocular axial biometry and refractive error, as well as age, should be matched between subgroups. Ocular biometry was performed with optical coherence tomography (OCT, model: REVO FC, Optopol Technology, Poland), that allows assessment of geometrical parameters of the eye on its axis, including axial length (AL), anterior chamber depth (ACD), intraocular lens thickness (LT), and central corneal thickness (CCT) expressed in millimeters. These parameters were used to ensure that the observed differences in measured visual parameters could be attributed to factors related to sport modality and expertise and not to differences in refractive error.

### Statistical analysis

No missing or outlying data, requiring specific handling, was recorded. Participants were divided into control group and sport shooters group; the latter being further divided into pistol shooters and rifle shooters subgroup. Additionally, to evaluate experts’ superiority, an additional subgroup of shooters from the Polish National Team were subdivided and compared with less experienced athletes. The Shapiro-Wilk test for normality was used to evaluate each dataset. Depending on the distribution of the two datasets, parametric or non-parametric tests were used to assess statistically significant differences between independent samples (subgroups). To account for potential confounding factors, age, refractive error, and ocular biometry were included in the analysis to ensure that observed differences in visual function were not attributable to these variables. Participants were age-matched between groups, and ocular biometry measures, including axial length, anterior chamber depth, and lens thickness, were analyzed to rule out their influence on vision parameters.

Statistical adjustments were applied as follows:

- Pearson’s correlation coefficients (R) were calculated to assess the relationship between age, shooting experience, and visual function variables.
- Multiple linear regression analysis was performed to determine whether shooting experience remained a significant predictor of visual acuity and contrast sensitivity after adjusting for age and ocular biometry.
- For categorical variables, chi-square tests were used to compare distributions of eye dominance and handedness across groups.

These statistical methods ensured that differences in visual functions were not solely explained by age-related decline or differences in ocular structure or refractive error.

Post hoc pairwise group comparisons were conducted using the Bonferroni correction to adjust for multiple comparisons within each visual function parameter.

## Results

Forty-nine participants (24 F/25 M) were recruited. One participant was rejected from participation due to high refractive error (-7.00 diopters) causing notable changes in the visual field. Therefore, forty-eight participants (24 F/24 M) were divided into three groups: rifle shooters (6 F/6 M), pistol shooters (8 F/8 M) and controls (10 F/10 M). A smaller proportion of rifle shooters required optical correction for distance viewing (41%) than pistol shooters (62%) and controls (50%), but this difference is reported to be statistically insignificant. All participants were right-handed. As some of the visual functions strongly correlate with age, care was taken to include participants of similar age in each subgroup. There were no statistically significant differences in age and gender distribution between any pair of the study subgroups compared; however, rifle shooting group was slightly older than the pistol shooting group (mean ± standard deviation: 27 ± 11 vs. 23 ± 8 y/o, *p* = 0.217) and therefore had slightly more years of experience in sports (*p* = 0.098). In Table 1 demographic and basic visual characteristics were summarized for each study subgroup.

**Table 1:** Demographic and visual characteristics for each of the study subgroups and p-values of independent samples test between each pair of the datasets.

	Control (n = 20, 10F/10M)	Pistol (n = 16, 8F/8M)	Rifle (n = 12, 6F/6M)	Shooters (n = 28, 14F/14M)	Control vs. Shooters	Rifle vs. Pistol	Control vs. Rifle	Control vs. Pistol
Age [y/o]								
Mean ± SD	25 ± 4	23 ± 8	27 ± 11	25 ± 9	0.457	0.217	0.907	0.212
Median	23.5	20.0	26	24				
Range	[19, 35]	[14, 40]	[16, 54]	[14, 54]				
Experience in shooting [years]								
Mean ± SD	N/A	9 ± 8	15 ± 10	12 ± 9	N/A	0.098	N/A	N/A
Median		5.5	12	11				
Range		[1, 26]	[4, 39]	[1, 39]				
Dominant eye [Right/Left]	13/7	15/1	12/0	27/1	0.005*	0.427	0.024*	0.044*
SE OD [Dptr]								
Mean ± SD	-0.60 ± 1.32	-0.64 ± 1.34	-0.08 ± 0.34	-0.30 ± 1.03	0.257	0.096	0.067	0.817
Median	-0.19	-0.19	0.00	0.00				
Range	[-3.38, 3.00]	[-5.13, 0.88]	[-0.75, 0.50]	[-4.75, 2.25]				
SE OS [Dptr]								
Mean ± SD	-0.81 ± 1.71	-0.52 ± 1.39	-0.08 ± 0.27	-0.22 ± 1.01	0.103	0.32	0.055	0.358
Median	-0.19	-0.13	0.00	0.00				
Range	[-5.38, 3.00]	[-5.25, 1.25]	[-0.50, 0.38]	[-4.75, 2.50]				
SE dominant eye [Dptr]								
Mean ± SD	-0.68 ± 1.43	-0.66 ± 1.34	-0.08 ± 0.34	-0.41 ± 1.06	0.285	0.073	0.07	0.882
Median	-0.19	-0.19	0.00	0.00				
Range	[-3.5, 3.0]	[-5.13, 0.88]	[-0.75, 0.50]	[-5.13, 1.25]				

*SD* standard deviation, *OD* oculus dexter, right eye, *OS* oculus sinister, left eye, *SE* Spherical equivalent of refractive correction,

*Statistical significance.

Table 2 summarizes the comparisons of monocular, binocular, and oculomotor visual functions across the control, pistol, and rifle groups. Shooters demonstrated significantly superior near visual acuity, higher contrast sensitivity (particularly in the dominant eye), and better Vernier acuity thresholds compared to controls. Partial η² values indicated small-to-moderate effect sizes for these differences.

**Table 2:** Visual acuity measures assessed in the study for each of the study subgroups and p-values of independent samples test between each pair of the datasets.

	Study subgroup				P-value, independent samples			
	Control (n = 20, 10F/10M)	Pistol (n = 16, 8F/8M)	Rifle (n = 12, 6F/6M)	Shooters (n = 28, 14F/14M)	Control vs. Shooters	Rifle vs. Pistol	Control vs. Rifle	Control vs. Pistol
BCVA OD [logMAR]								
Mean ± SD	-0.12 ± 0.09	-0.15 ± 0.05	-0.09 ± 0.12	-0.13 ± 0.09	0.760	0.091	0.495	0.186
Median	-0.10	-0.15	-0.10	-0.14				
Range	[-0.28, 0.06]	[-0.22, -0.05]	[-0.28, 0.08]	[-0.28, 0.08]				
BCVA OS [logMAR]								
Mean ± SD	-0.10 ± 0.10	-0.15 ± 0.07	-0.13 ± 0.11	-0.14 ± 0.09	0.182	0.554	0.514	0.120
Median	-0.10	-0.16	-0.16	-0.16				
Range	[-0.28, 0.06]	[-0.30, -0.04]	[-0.30, 0.04]	[-0.30, 0.04]				
BCVA OU [logMAR]								
Mean ± SD	-0.18 ± 0.09	-0.20 ± 0.07	-0.13 ± 0.10	-0.17 ± 0.09	0.633	0.033*	0.122	0.510
Median	-0.19	-0.19	-0.09	-0.16				
Range	[-0.3, 0.00]	[-0.30, -0.09]	[-0.28, 0.00]	[-0.30, 0.00]				
BCVA Difference [logMAR]								
Mean ± SD	0.04 ± 0.04	0.06 ± 0.05	0.12 ± 0.06	0.09 ± 0.06	0.007**	0.013**	0.001**	0.206
Median	0.04	0.05	0.11	0.08				
Range	[0.00, 0.12]	[0.00, 0.20]	[0.03, 0.24]	[0.00, 0.24]				
Better eye: Dominant/Other/Equal	10-05-2005	08-06-2002	04-08-2000	12/14/2	0.041*	0.101	0.012**	0.305
Near BCVA OD [logMAR]								
Mean ± SD	0.03 ± 0.07	-0.02 ± 0.07	-0.07 ± 0.05	-0.04 ± 0.07	0.003**	0.032*	<0.001**	0.084
Median	0.00	-0.02	-0.05	-0.06				
Range	[-0.01, 0.12]	[-0.1, 0.10]	[-0.18, 0.00]	[-0.18, 0.10]				
Near BCVA OS [logMAR]								
Mean ± SD	0.01 ± 0.07	-0.02 ± 0.08	-0.06 ± 0.05	-0.03 ± 0.07	0.015**	0.127	0.003**	0.176
Median	0.00	-0.01	-0.06	-0.06				
Range	[-0.01, 0.12]	[-0.18, 0.10]	[-0.14, 0.02]	[-0.18, 0.10]				
Near BCVA OU [logMAR]								
Mean ± SD	-0.04 ± 0.09	-0.06 ± 0.06	-0.10 ± 0.07	-0.08 ± 0.06	0.102	0.099	0.029*	0.278
Median	-0.02	-0.07	-0.10	-0.08				
Range	[-0.16, 0.16]	[-0.18, 0.00]	[-0.20, 0.00]	[-0.20, 0.00]				
Near BCVA difference [logMAR]								
Mean ± SD	0.02 ± 0.05	0.04 ± 0.04	0.02 ± 0.03	0.03 ± 0.04	0.201	0.539	0.459	0.187
Median	0.00	0.02	0.01	0.02				
Range	[-0.1, 0.13]	[0.00, 0.12]	[0.00, 0.08]	[0.00, 0.12]				
Vernier VA OD [‘’]								
Mean ± SD	11.1 ± 6.3	5.6 ± 3.9	8.0 ± 6.4	6.6 ± 5.1	0.010**	0.341	0.019*	0.004**
Median	11.5	4.3	6.4	5.2				
Range	[1.8, 22.7]	[1.5, 12.6]	[1.8, 19.6]	[1.5, 19.6]				
Vernier VA OS [‘’]								
Mean ± SD	16.9 ± 10.8	8.8 ± 6.8	7.8 ± 4.8	8.4 ± 5.9	0.001**	0.651	0.010**	0.013**
Median	14.4	7.8	7.3	7.3				
Range	[2.4, 38.2]	[1.5, 12.6]	[1.5, 17.0]	[0.5, 19.6]				
Vernier VA OU [‘’]								
Mean ± SD	8.7 ± 5.1	5.0 ± 3.2	5.8 ± 3.3	5.4 ± 3.2	0.032*	0.505	0.029*	0.018*
Median	7.8	5	4.7	4.8				
Range	[1.3, 17.0]	[1.5, 11.6]	[1.6, 11.3]	[1.5, 11.6]				
Better eye: Dominant/Other/Equal	10-10-2000	09-07-2000	07-05-2000	16-12-2000	0.637	0.935	0.669	0.727
Contrast sensitivity OD (logCS)								
Mean ± SD	1.74 ± 0.07	1.79 ± 0.08	1.80 ± 0.08	1.80 ± 0.08	0.005**	0.924	0.022*	0.009**
Median	1.74	1.84	1.78	1.8				
Range	[1.64, 1.84]	[1.56, 1.88]	[1.68, 1.94]	[1.56, 1.94]				
Contrast sensitivity OS (logCS)								
Mean ± SD	1.78 ± 0.05	1.73 ± 0.12	1.74 ± 0.08	1.74 ± 0.10	0.277	0.688	0.127	0.691
Median	1.76	1.76	1.72	1.76				
Range	[1.72, 1.88]	[1.52, 1.84]	[1.64, 1.88]	[1.52, 1.88]				
Contrast sensitivity OU (logCS)								
Mean ± SD	1.80 ± 0.06	1.80 ± 0.10	1.83 ± 0.12	1.81 ± 0.10	0.229	0.228	0.103	0.627
Median	1.8	1.8	1.83	1.81				
Range	[1.72, 1.92]	[1.52, 1.92]	[1.52, 1.92]	[1.50, 1.92]				
CS difference (logCS)								
Mean ± SD	0.01 ± 0.07	0.07 ± 0.09	0.05 ± 0.07	0.06 ± 0.08	0.019*	0.631	0.102	0.028*
Median	0.00	0.04	0.04	0.04				
Range	[-0.12; 0.12]	[0.00, 0.34]	[0.00, 0.24]	[0.00, 0.34]				

*BCVA* best corrected visual acuity for distance, *OD* oculus dexter, right eye, *OS* oculus sinister, left eye, *OU* Oculi uterque – both eyes, *CS* contrast sensitivity, *VA* visual acuity, *DOM* dominant eye.

*Statistical significance, **Statistically significant difference after applying Bonferroni correction.

**Fig. 2:**
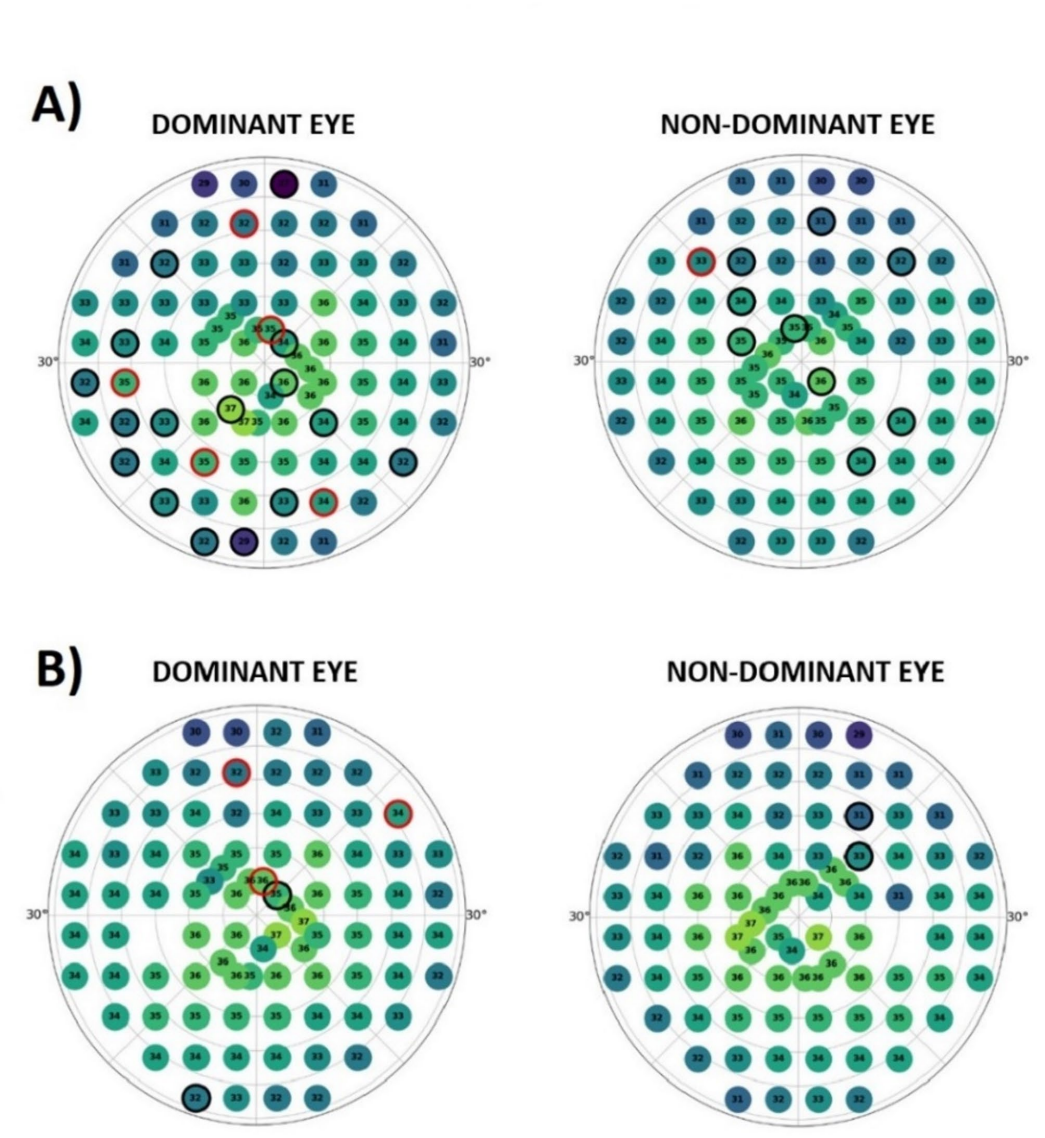
Color-coded perimetric maps for (**a**) pistol and (**b**) the rifle shooter subgroup. Numerical values represent median values of the sensitivity in decibels [dB] at each point on the visual field. Black circles denote points at which results were significantly lower than in the control group, and red circles denote areas in which results were significantly higher than in the control group.

Statistically significant difference in the perimetric measures other than eye movements and sensitivity maps was noted in right eye HoV@10° [dB] which parameter represents the peak retinal sensitivity of the eye. Statistically significant difference was noted between rifle and pistol shooter, with the latter group achieving superior results (35.7 ± 1.7 dB vs. 34.2 ± 1.7 dB, *p* = 0.029). Data were limited to participants who were right-eyed dominant, which enables direct comparison of perimetric maps of dominant and non-dominant eyes. No statistically significant differences between any of the subgroups in any of the perimetry reliability measures were noted, enabling direct comparison between subgroups. Color-coded perimetric maps with median values of sensitivity in decibels for pistol shooters and rifle shooters were displayed below (Fig. 2).

Ocular biometry measures for each of the study subgroups were summarized in (Table 3).

**Table 3:** Mean, standard deviations, medians, and ranges for ocular biometry parameters for each of the study subgroups and p-values of independent samples test.

	Study subgroup				P-value, independent samples			
	Control (n = 20, 10F/10M)	Pistol (n = 16, 8F/8M)	Rifle (n = 12, 6F/6M)	Shooters (n = 28, 14F/14M)	Control vs. Shooters	Rifle vs. Pistol	Control vs. Rifle	Control vs. Pistol
AL OD [mm]								
Mean ± SD	24.20 ± 1.34	23.66 ± 0.83	23.76 ± 0.72	23.69 ± 0.78	0.185	0.811	0.458	0.235
Median	23.99	23.79	23.74	23.79				
Range	[21.25, 25.91]	[22.32,24.98]	[22.53, 24.54]	[22.32, 24.98]				
AL OS [mm]								
Mean ± SD	24.24 ± 1.38	23.47 ± 0.81	23.70 ± 0.57	23.57 ± 0.73	0.081	0.600	0.368	0.112
Median	24.08	23.37	23.84	23.62				
Range	[21.28, 26.04]	[22.30, 24.73]	[22.65, 23.21]	[22.32, 24.77]				
ACD OD [mm]								
Mean ± SD	3.41 ± 0.28	3.67 ± 0.32	3.41 ± 0.31	3.58 ± 0.33	0.126	0.123	0.993	0.036*
Median	3.32	3.62	3.46	3.59				
Range	[3.02, 3.83]	[3.19, 4.13]	[2.92, 3.79]	[2.92, 4.13]				
ACD OS [mm]								
Mean ± SD	3.46 ± 0.22	3.69 ± 0.27	3.49 ± 0.35	3.62 ± 0.30	0.092	0.205	0.798	0.023*
Median	3.42	3.63	3.51	3.58				
Range	[2.99, 3.86]	[3.27, 4.06]	[2.94, 3.90]	[2.94, 4.06]				
LT OD [mm]								
Mean ± SD	3.94 ± 0.67	3.55 ± 0.23	3.75 ± 0.38	3.61 ± 0.29	0.052	0.175	0.505	0.030*
Median	3.64	3.52	3.61	3.54				
Range	[3.50, 5.53]	[3.21, 3.89]	[3.39, 4.41]	[3.21, 4.41]				
LT OS [mm]								
Mean ± SD	3.72 ± 0.19	3.57 ± 0.22	3.49 ± 0.23	3.54 ± 0.22	0.018*	0.508	0.029*	0.023*
Median	3.68	3.51	3.48	3.5				
Range	[3.41, 4.17]	[3.26, 3.94]	[3.25, 3.89]	[3.25, 3.94]				
CCT OD [µm]								
Mean ± SD	547 ± 32	543 ± 36	553 ± 38	547 ± 36	0.985	0.595	0.693	0.843
Median	550	540	540	540				
Range	[510, 590	[490, 610]	[510, 610]	[490, 610]				
CCT OS [µm]								
Mean ± SD	553 ± 28	548 ± 33	558 ± 47	551 ± 37	0.851	0.577	0.765	0.625
Median	560	550	570	550				
Range	[510, 590]	[500, 610]	[480, 610]	[0.480, 0.610]				

*OD* Oculus Dexter – right eye, *OS* Oculus Sinister – left eye, *AL* Axial length, *ACD* anterior chamber depth, *LT* lens thickness, *CCT* central corneal thickness.

*Statistically significant, **Statistically significant difference after applying Bonferroni correction.

Eye movement types as displayed on the gaze shift diagram were divided based on the methodology described earlier into basic movements and gaze shifts, the latter including small, average, and large gaze shifts and fixation losses. Data were summarized in (Table 4). Proportion of time each individual gazes within and outside of the region defining the target is displayed in Table 4 as Basic movements and Gaze shifts, respectively.

While the need for vision correction does not disqualify participants from participating in shooting competitions or owning a gun, refractive error may influence other ocular measures assessed in the study. Therefore, the spherical equivalents (SE OD and SE OS) of the habitual refractive corrections were compared between each pair of study subgroups. Although the shooting group and rifle shooters were on average less myopic than their nonathletic peers, the difference in SE was not statistically significant (Table 1). In addition, no statistically significant difference in axial length of the eye globe between any pair of datasets from each subgroup additionally supports the lack of difference in refractive error of the participants, since the axial length strongly correlates with the refractive error. Therefore, other visual parameters that depend on refraction (like the near point of accommodation or visual acuity) could be directly compared.

**Table 4:** Averages, standard deviations, medians, and ranges of gaze shift diagram parameters for each of the study subgroups and p-values of independent samples test.

	Study subgroup				P-value, independent samples			
	Control (n = 20, 10F/10M)	Pistol (n = 16, 8F/8M)	Rifle (n = 12, 6F/6M)	Shooters (n = 28, 14F/14M)	Control vs. Shooters	Rifle vs. Pistol	Control vs. Rifle	Control vs. Pistol
Basic movements [%]								
Mean ± SD	93.8 ± 4.2	96.0 ± 4.1	96.1 ± 5.7	96.0 ± 4.8	0.035*	0.706	0.049*	0.099
Median	95.0	97.5	98	98.0				
Range	[87.0, 100]	[87.0, 100]	[81.0, 100]	[81.0, 100]				
Gaze shifts [%]								
Mean ± SD	6.2 ± 4.2	4.0 ± 4.1	3.9 ± 5.7	4.0 ± 4.8	0.035*	0.706	0.049*	0.099
Median	5.0	2.5	2.0	2.0				
Range	[0.0, 13.0]	[0.0, 13.0]	[0.0, 19.0]	[0.0, 19.0]				
Small gaze shifts [%]								
Mean ± SD	4.5 ± 4.0	3.1 ± 3.3	3.5 ± 5.5	3.3 ± 4.3	0.163	0.686	0.199	0.272
Median	3.0	2.0	2.0	2.0				
Range	[0.0, 12.0]	[0.0, 10.0]	[0.0, 19.0]	[0.0, 5.0]				
Average gaze shifts [%]								
Mean ± SD	1.5 ± 2.3	0.9 ± 1.4	None	0.8 ± 1.1	0.015**	0.537	0.015**	0.128
Median	1.0	0.5		0.0				
Range	[0.0, 4.0]	[0.0, 5.0]		[0.0, 5.0]				
Large gaze shifts [%]								
Mean ± SD	0.13 ± 0.35	None	None	None	0.055*	N/A	0.221	0.150
Median	0.00							
Range	[0.00, 1.00]							
Fixation losses [%]								
Mean ± SD	None	0.06 ± 0.25	None	0.04 ± 0.19	0.495	0.427	N/A	0.366
Median		0.00		0.00				
Range		[0.00, 1.00]		[0.00, 1.00]				
Ratio - first half								
Mean ± SD	0.05 ± 0.04	0.05 ± 0.05	0.04 ± 0.01	0.04 ± 0.05	0.140	0.508	0.161	0.258
Median	0.04	0.02	0.01	0.02				
Range	[0.00, 0.09]	[0.00, 0.16]	[0.00, 0.10]	[0.00, 0.16]				
Ratio - second half								
Mean ± SD	0.09 ± 0.09	0.04 ± 0.05	0.07 ± 0.14	0.05 ± 0.09	0.018*	0.621	0.032*	0.042*
Median	0.05	0.03	0.03	0.03				
Range	[0.00, 0.31]	[0.00, 0.14]	[0.00, 0.48]	[0.00, 0.48]				

*Statistically significant difference, **Statistically significant difference after applying Bonferroni correction.

No statically significant differences were noted in heterophorias for distance and near, stereoacuity, near point of accommodation, convergence and renewal and accommodation facility between any subgroup (Table 5).

**Table 5:** Binocular vision and accommodative function parameters assessed in the study for each of the study subgroups and p-values of independent samples test between each pair of datasets.

	Study subgroup				P-value, independent samples			
	Control (n = 20, 10F/10M)	Pistol (n = 16, 8F/8M)	Rifle (n = 12, 6F/6M)	Shooters (n = 28, 14F/14M)	Control vs. Shooters	Rifle vs. Pistol	Control vs. Rifle	Control vs. Pistol
Horizontal heterophorias for distance* [Δ]								
Mean ± SD	-0.1 ± 2.5	0.0 ± 3.9	-0.2 ± 1.4	-0.1 ± 3.1	0.899	0.527	0.581	0.810
Median	0.3	0.5	0.2	0.5				
Range	[-9.0, 5.0]	[-9.0, 9.0]	[-4.0, 1.0]	[-9.0, 9.0]				
Type: ESO/EXO/ORTHO	10/8/1	9/6/1	6/5/1	15/11/2	-	-	-	-
Horizontal heterophorias for near* [Δ]								
Mean ± SD	-1.5 ± 3.3	-1.3 ± 5.1	-2.2 ± 4.0	-1.7 ± 4.6	0.888	0.649	0.625	0.899
Median	-1.3	-2.0	-1.0	-1.50				
Range	[-3.0, 6.0]	[-10.0, 9.0]	[-11.0, 3.0]	[-11.0, 9.0]				
Type: ESO/EXO/ORTHO	5/13/2	5/11/0	3/9/0	8/20/0	-	-	-	-
Stereoacuity [‘’]								
Mean ± SD	30 ± 10	32 ± 12	26 ± 7.5	29 ± 11	0.559	0.278	0.234	0.999
Median	25	25	25	25				
Range	[20, 50]	[20, 60]	[20, 40]	[30, 60]				
Near point of accommodation OU [cm]								
Mean ± SD	10.6 ± 2.4	11.4 ± 3.6	11.3 ± 4.5	11.3 ± 3.9	0.608	0.987	0.521	0.414
Median	11.0	11.5	9.4	11.0				
Range	[6.0, 14.5]	[6.3, 20.8]	[6.7, 21.0]	[6.3, 21.0]				
Near point of convergence OU [cm]								
Mean ± SD	6.4 ± 3.4	8.3 ± 4.3	8.2 ± 4.1	8.3 ± 4.0	0.256	0.690	0.624	0.140
Median	7.2	8.8	6.6	7.1				
Range	[4.7, 12.3]	[0.0, 16.7]	[5.0, 16.0]	[0.0, 16.7]				
Point of renewal OU [cm]								
Mean ± SD	11.6 ± 2.4	12.4 ± 3.6	12.4 ± 3.6	12.4 ± 3.9	0.706	0.978	0.563	0.460
Median	11.0	12.5	12.5	23.0				
Range	[8.0, 17.0]	[7.3, 21.8]	[7.3,21.8]	[7.3, 22.0]				
Accommodation facility OU [cpm]								
Mean ± SD	11.1 ± 2.9	10.7 ± 4.1	11.6 ± 2.8	11.1 ± 3.5	0.977	0.519	0.652	0.726
Median	10.5	9.5	12	10.5				
Range	[4.0, 24.0]	[4.0, 18.0]	[8.0, 18.0]	[4.0, 18.0]				
Accommodation facility OD [cpm]								
Mean ± SD	12.8 ± 5.2	12.4 ± 4.1	15.0 ±3.6	13.4 ± 4.0	0.694	0.219	0.333	0.853
Median	12.5	12.0	15.0	13.0				
Range	[4.0, 24]	[7.0, 20.0]	[10.0, 20.0]	[7.0, 20.0]				
Accommodation facility OS [cpm]								
Mean ± SD	13.1 ± 6.5	10.2 ± 3.8	13.3 ± 3.8	11.4 ± 4.0	0.359	0.132	0.935	0.205
Median	12.0	9.5	12.0	12.0				
Range	[4.0, 30.0]	[5.0, 17.0]	[9.0, 20.0]	[5.0, 20.0]				

*OD* Oculus Dexter, right eye, *OS* Oculus Sinister, left eye, *OO* Oculi Uterque – both eyes, *cpm* cycles per minute, *Dptr* diopters; pdptr – prism diopters.

*Exophoria < 0 pdptr, esophoria > 0 pdptr; ESO – esophoria; EXO – exophorias; ORTHO – orthophoria.

### Experts’ superiority

To additionally evaluate experts’ superiority, shooters were further divided into participants who were training to be sport shooters and members of the National Shooting Team. The latter group included 10 rifle shooters and 5 pistol shooters. Statistically significant differences between the two subgroups were found in near visual acuity of both monocular eyes and of the dominant eye, with National Team members having superior visual acuity. This is especially interesting considering the statistically significant difference in age between these two groups, with National Team Members being older (29 vs. 18 y/o). Since near vision is deteriorating with increasing age, the age difference should work on the National Team members’ disadvantage. Additionally, Members of the National Team show significantly fewer eye shifts during testing (*p* = 0.044, 2.1% vs. 6.1%), significantly more stable gaze during the first half of the perimetric recording (*p* = 0.033, ratio of 0.023 vs. 0.061), and better stereoacuity.

(p = 0.005, 25‘’ vs. 35’’) than the rest of the shooting group. The one parameter in which the National Team members are having worse results than the other subgroup is the sensitivity in some parts of the visual field; however, these differences cannot be fully attributed to their shooting performance, due to the significant difference in age between both of these groups, which most probably led to older participants having lower sensitivity thresholds than less experienced, younger athletes.

### Linear correlations

Statistically significant linear correlations were observed between age and the near point of accommodation (*r* = 0.542, *p* < 0.001), point of renewal (*r* = 0.537, *p* < 0.001), ratio of gaze shifts during the first half of the measurements (*r* = -0.434, *p* = 0.002) and percentages of small and average gaze shifts (*r* = -0.033, *p* = 0.022 and *r* = -0.309, *p* = 0.033, respectively). Vernier acuities were negatively linearly correlated with the level of participant shooting experience, but not with age (*r* = -0.327, *p* = 0.023; *r* = -0.298, *p* = 0.039 and − 0.319, *p* = 0.027 for the dominant eye, right eye, and left eye, respectively). Other parameters that were significantly linearly correlating with participants’ experience, but not with age, were the NVA of the shooting eye (*r* = -0.486, *p* < 0.001), binocular NVA (*r* = -0.306, *p* = 0.034), contrast sensitivity of the dominant eye (*r* = 0.361, *p* = 0.011), which three were improving with increasing experience and NPC (*r* = 0.335, *p* = 0.020) which was worsening with experience.

In a multivariate regression model the top five independent variables that have the strongest relation with shooting experience were the NVA of the dominant eye, binocular NVA, difference between eye in near visual acuity, contrast sensitivity of the non-dominant eye and the difference in contrast sensitivity between eyes.

## Discussion

In a study examining visual performance in skeet shooters, visual functions of experts were not found to be above normal^34^. Therefore, research suggested that attempts to improve shooting performance by training general sight characteristics could be counterproductive. However, critical differences between skeet shooting and precision shooting modalities do not allow these observations to be directly applied to the latter. This study compares basic visual functions in participants regularly practicing precision shooting (both air pistol and air rifle modalities) with age-, gender- and refractive error-matched controls.

Notably, not all athletes reached the normal BCVA of 0.00 logMAR. This may imply that exceptional visual acuity and emmetropia are not necessary for shooting performance. When it comes to differences in **best corrected visual acuities** for distance, a statistically significant difference was observed between rifle and pistol shooting athletes, with pistol shooting group having superior BCVA (*p* = 0.033). This superiority is further supported by the fact that the central visual field sensitivity thresholds are significantly higher in pistol shooters (especially in their dominant eye) and by their peak central visual field sensitivity of the dominant eye compared to rifle shooting group (HoV@10° [dB]: 35.7 ± 1.7 vs. 34.2 ± 1.7, *p* = 0.029). Interestingly, in the rifle modality subgroup the eye with higher BCVA was more often the non-dominant eye, whereas an opposite trend was observed in the control group. Also, in the rifle subgroup, visual acuities of both eyes were rarely equal, showing higher disproportions between the dominant (aiming) and non-dominant (covered) eye. What is interesting is that in these athletes the dominant eye for distance also had the best NVA, which may be due to the monocular training that these athletes perform. Precision shooters often aim with one eye covered to prevent diplopia, resulting in habitual monocular viewing that may affect binocular vision over time. During such focus-demanding monocular training the dominant eye for distance may take over the role of the dominant eye for near vision. Rifle shooting group being slightly (insignificantly) older than the other subgroups which should work on their disadvantage when it comes to visual acuity at near, and even though rifle shooters excel in NVA. Furthermore, in the shooting group, the NVA of the shooting eye (*r* = -0.486, *p* < 0.001) and the binocular NVA (*r* = -0.306, *p* = 0.034) were correlating with experience, but not with age, further supporting this observation. Taking all of this into account, we may describe pistol shooters as more distance-oriented, while the rifle shooters seem to be more focused on the near work with the dominant eye for distance taking over the functions of the dominant eye for near at the expense of their peak visual acuity and contrast sensitivity at distance. Although statistically significant differences in visual acuity were observed between groups, it is important to note that these differences were relatively small and likely fall within the expected test–retest variability for optotype-based visual acuity. Therefore, their clinical relevance may be limited, and these results should be interpreted with caution when considering their potential impact on everyday visual function. Furthermore, as the sample size calculation was based on Vernier acuity thresholds rather than standard VA, the study was not specifically powered to detect subtle group differences in VA, and these results should therefore be interpreted with caution. Even though for the rifle shooters group the difference in BCVA between both eyes (0.12 logMAR) is statistically and clinically significant. Comparing BCVA between the dominant and non-dominant eye yields similar results to comparing the left and right eyes.

It should be noted that the Miles test and the fogging technique, used in the study to classify eye dominance in do not quantify the degree of dominance. The stronger right-eye dominance observed among shooters may reflect a self-selection effect, where individuals with stronger or clearer sighting dominance are more likely to excel in precision sports that rely on consistent monocular aiming. Research shows that there is no correlation among hand laterality and achieving ocular domination in the targeted sports^35^, however future research could benefit from incorporating measures that assess the magnitude of sensory or motor dominance.

**Perimetric sensitivity maps** revealed that rifle shooters are characterized by lower retinal sensitivity than pistol shooters and controls in many parts of the central visual field. This may imply that peak sensitivity in the visual field may not be the key factor in rifle shooting or may even deteriorate with training. On the other hand, pistol shooters exhibit superior sensitivity thresholds in the central part of their visual field and have higher peak sensitivity values for the dominant eye than other subgroups. This supports the hypothesis that pistol shooters and rifle shooters may differ in their visual needs, pistol shooters being more focused on distance and rifle shooters on near visual tasks. One could argue that these differences can be attributed to a slight (insignificant) difference in age between these subgroups However, adding to that higher BCVA and higher HoV@10° in pistol shooters and no significant difference in refractive error and ocular geometry between any of these subgroups, these changes are more likely related to the type of shooting modality and training than to slight differences in age.

Even though the differences in retinal sensitivity across many parts of the visual field were statistically significant (even after applying the Bonferroni correction), these differences fell within the typical interobserver variability and may not be clinically meaningful. However, it is important to note that the threshold strategy applied in this study allows retinal sensitivity to be pinpointed with a high level of accuracy (± 1 dB). Using this strategy, test–retest variability for healthy individuals in the central 30 degrees of the visual field is approximately ± 1 to 2 dB at individual points. Although a 2 dB difference at a single test location should be interpreted with caution, as it may not be clinically meaningful, a consistent 2 dB difference in mean sensitivity across multiple test points — as observed in rifle shooters compared to non-athletes — could potentially be clinically significant. Fixational eye movements help prevent Troxler’s effect (peripheral fading). The study reported reduced retinal sensitivity and more stable fixation in shooters. Therefore, the decreased retinal sensitivity observed could be linked to ‘quiet eye’ behavior.

The shooters also have superior **contrast sensitivity of the dominant eye** compared to controls. It should be noted that most participants had higher than average peak contrast sensitivity (> 1.62 log CS (0.06 SD))^36^, which suggests that none of the participants had severe vision deficiencies. Shooters of both modalities were also reported to have significantly larger differences in logCS between the two eyes, with the dominant eye being significantly better than the non-dominant eye, which further supports that there exists an imbalance in visual parameters in shooters, who routinely cover one eye during aiming to prevent diplopia. Comparing Contrast sensitivity between the dominant and non-dominant eye yields similar results to comparing the left and right eyes.

Significant statistical differences in all measures of **Vernier acuity** were reported between any subgroup of shooting athletes and the control group, with the shooting group achieving significantly better results.

This may point to this subgroup’s superior cortical functions compared to controls. Vernier tasks are practiced regularly by these athletes; therefore, these differences could be attributed to training. Furthermore, Vernier acuities were negatively correlated with shooting experience, but not with age, which indicates that these parameters are superior in more experienced athletes and may improve with training.

Even though **accommodation** is reported as a critical factor during aiming, shooters’ ability to use or relax their accommodation was not reported to be better than noted in the control group. This may be since the rear sight and front sights are much further away from the athlete’s eye than their NPA and therefore aiming requires using only a fraction of their accommodative amplitude, or perhaps training does not create an environment for accommodation training and opportunity to improve it with time. In each subgroup at least one participant with lower-than-average binocular accommodation facility was reported. This may imply that accommodative performance is not influenced by shooting training and changes gradually with age regardless of the shooting modality. Some studies show that accommodation facility could be improved with proper training.^33^.

It is known that **quiet eye** can accelerate the acquisition of sporting skills and is associated with an improvement in eye–hand coordination.^7^. Research demonstrates prolonged quiet eye periods as an effective marker for differentiating skilled and less-skilled athletes^37,38^. Training programs with elite skeet shooters have shown earlier quiet eye onset and longer duration, improving their performance^39^. This study shows that shooting athletes have significantly less gaze shifts during perimetry recording than the control group and have superior ability to maintain quiet eye state during focus-demanding tasks. Shooters were shown to spend significantly more time focusing within the region defining the target than controls (96,0 vs. 93.8%, *p* = 0.035). Significantly lower ratios of gaze shifts in the second half of the perimetric recording show that shooters maintain focus for longer and are not as prone to visual fatigue, as age-matched controls. Shooters demonstrated more stable gaze patterns with fewer gaze shifts aligns with the well-documented ‘quiet eye’ phenomenon observed in elite performers during precision tasks. Previous studies have shown that the rate of microsaccades decreases during tasks requiring intense visual concentration and fine motor control, such as shooting^40^, golf putting^41^, or threading a needle^42^. This reduced microsaccadic activity is thought to reflect enhanced top-down attentional control and may help maintain high spatial resolution by minimizing retinal image motion. Our results support the notion that trained shooters develop improved fixation stability, which likely contributes to their performance advantage.

Fixational eye movements such as microsaccades are known to counteract Troxler’s fading by continually refreshing retinal stimulation, particularly in the peripheral field. Therefore, the reduced peripheral retinal sensitivity observed in this study in shooters may be partially explained by their more stable fixation behavior (‘quiet eye’). In highly trained shooters, decreased fixational eye movements may therefore result in less stimulation of peripheral photoreceptors during static perimetry, contributing to the lower sensitivity values observed in more experience shooters.

When it comes to **experts’ superiority**, the National Team members have superior NVA both monocularly and binocularly. This is especially interesting considering that National Team Members are significantly older (29 vs. 18 y/o) than other athletes. Members of the National Team also show significantly fewer eye movements during testing, more stable fixation during the first half of the perimetric recording, and better stereoacuity than the rest of the athletes, which may suggest that experts can more easily achieve and maintain focus. Not all parameters, however, improved with growing experience: as in both rifle shooting group and National Team Members lower eye sensitivity in some parts of the visual field were reported compared with controls. Additionally, the near point of convergence seems to worsen with experience, pointing to further disruption of binocular vision and decreasing balance with training.

When it comes to **biometry measures**, there are no statistically significant differences in eye axial length between any pair of study subgroups which supports the lack of significant difference in refractive errors between subgroups. This is important, as some parameters may depend on the refractive error. Most comparisons did not reveal statistically significant differences; notable exceptions included the anterior chamber depth (ACD OD and OS) and lens thickness (LT OD and OS) measurements. These small differences likely reflect normal inter-individual variability in ocular biometry that may persist even when groups are matched for age and spherical equivalent refraction. Additionally, factors such as measurement conditions (e.g., accommodation during biometry) and small differences in axial length, which were not significantly different, could contribute to variation in ACD and LT. Importantly, these biometric differences were accounted for in the statistical analysis to ensure that they did not confound the comparison of functional vision parameters between groups. The lens being thicker in the control groups than in shooting group may suggest a more relaxed accommodative state in the latter group. Slightly lower pupil diameter in the control group supports this hypothesis, as pupil diameter decreases with increasing accommodation.

### Limitations

An important limitation of this cross-sectional study is that it cannot establish causality. The superior visual functions observed in shooters may reflect the effects of extensive sport-specific training; however, it is equally possible that individuals with naturally superior visual abilities are more likely to excel in and persist with precision shooting. A bidirectional relationship is also plausible. However, differences observed between less and more experienced shooters, linear correlations of ocular parameters with shooting experience and some detrimental effects observed among more experience shooters do not allow to fully disprove the conclusion that some differences can be attributed to shooting training.

An additional limitation of this study is that the subgroup comparisons within the shooter group (e.g., more experienced versus less experienced shooters) involved relatively small sample sizes, which may have limited the statistical power to detect subtle differences and increased the possibility of Type II errors. Although primary analyses were adequately powered based on effect size estimates for Vernier acuity, these post hoc subgroup findings should therefore be interpreted with caution. Nevertheless, this study is, to our knowledge, the first to compare elite-level precision shooters with age- and refraction-matched controls, providing a detailed profile of visual function in this specialized population. While a small number of prior studies have employed larger samples (*n* = 100, *n* = 40) - these studies generally focused on broader military populations rather than elite precision athletes or did not include properly matched control group^43,44^. Similarly, research on visually impaired shooters (*n* = 10–25, *n* = 19) has emphasized the critical role of contrast sensitivity in performance classification^45,46^. Taken together, these existing datasets support the relevance of visual acuity, contrast sensitivity, and stereoacuity to shooting performance and highlight the need for future studies with larger, well-matched samples to confirm and extend these subgroup results. Future longitudinal or training studies are needed to clarify the relative contributions of innate visual abilities and experience-dependent plasticity.

Also, it should be noted that the built-in gaze tracker used in this study has lower spatial resolution than dedicated eye-tracking systems and does not include additional calibration beyond standard perimetry alignment, which may introduce some variability when comparing gaze data across participants. Research has highlighted that gaze trackers incorporated in different devices cannot be used as good reliability measures for visual field testing^47^. However, in this study, we confirmed that all standard perimetry reliability measures were comparable between subgroups and no significant differences were found for these measures, enabling valid comparisons of perimetry results. The gaze behavior results remained statistically significant even after applying multiple comparison corrections, supporting the robustness of the observed group differences in fixation stability. However, more studies are needed to validate the use of perimetry in-built gaze-tracking system in eye movement analysis.

## Conclusions

Athletes outperform their peers in near and distance visual acuity, contrast sensitivity, and focus maintenance. National-level athletes exhibit greater gaze stability and reduced visual fatigue, reinforcing the role of quiet eye in training. However, some visual adaptation may lead to binocular vision imbalances, particularly in rifle shooting group, who develop a stronger dominant eye for near tasks at the expense of peak acuity and contrast sensitivity for distance. Differences in visual skills between shooting modalities highlight the need for customized visual training. Rifle shooters excel in near tasks, while pistol shooters have superior distant acuity and visual field sensitivity. These adaptations likely stem from long-term monocular training rather than natural visual superiority. Future research should investigate the long-term impact of shooting training and develop tailored interventions to optimize performance while minimizing potential negative adaptations.

## Practical implications

- Precision shooters develop enhanced visual acuity and stability, suggesting that targeted visual training could further refine their skills and improve performance.
- Pistol shooters excel in distance vision, while rifle shooters develop stronger near-focusing abilities.
- Training programs should be tailored to these specific visual demands.
- Long-term shooting practice may cause dominance-related imbalances between the eyes, potentially impacting performance. Regular vision assessments and corrective strategies can help mitigate these effects.
- National-level shooters exhibit superior eye stability and reduced fatigue. Training methods that reinforce quiet eye techniques and sustained focus could be beneficial for all skill levels.
- Regular eye check-ups and personalized vision training can help shooters maintain peak visual abilities and prevent potential strain or degradation over time, ensuring long-term performance sustainability.

## Data Availability

The data that support the findings of this study are available from the corresponding author upon reasonable request.
